# Tin–carbon nanomaterial formation in a helium atmosphere during arc-discharge

**DOI:** 10.1039/c9ra05485e

**Published:** 2019-11-11

**Authors:** Alexey Zaikovskii, Sergey Novopashin, Vasiliy Maltsev, Tatyana Kardash, Inna Shundrina

**Affiliations:** Kutateladze Institute of Thermophysics SB RAS Ac. Lavrentiev Ave. 1 630090 Novosibirsk Russia zale@itp.nsc.ru; Boreskov Institute of Catalysis SB RAS Ac. Lavrentiev Ave. 5 630090 Novosibirsk Russia; N. N. Vorozhtsov Novosibirsk Institute of Organic Chemistry SB RAS Ac. Lavrentiev Ave. 9 630090 Novosibirsk Russia

## Abstract

Electric arc discharge is a straightforward and attractive method for the synthesis of nanomaterials with unique properties. When electric arc sputtering of a composite tin–carbon electrode occurs in an inert gas medium, a material is formed that is composed of spherical tin nanoparticles surrounded by a carbon shell. The tin nanoparticles themselves have a core–shell structure with crystalline core and amorphous shell. Such a tin material has four times reduced enthalpy of melting due to the presence of an amorphous phase. However, the processes leading to the formation of nanostructures are not fully understood and require additional research. The collection of material at different distances from the arc discharge allows the identification of the processes leading to the formation of this structure. A mixture of carbon and tin vapours formed by electric arc sputtering forms a fan-shaped jet from the interelectrode gap, the temperature of which decreases with the distance from the discharge axis. Cooling the mixture leads to carbon condensation, and then tin condensation begins to occur on the carbon structures. Agglomeration of carbon-tin clusters and coagulation of tin leads to the formation of liquid tin nanoparticles coated with a carbon shell. The cooling of this material leads to the solidification of the tin and the transformation of the carbon shell. Different rates of cooling of the shell and the core of the tin particle lead to the formation of a core–shell structure with an amorphous shell and a crystalline core.

## Abstract 2

Elongated carbon shells partially filled with tin, spherical carbon shells partially filled with tin, and chains of spherical tin particles coated with a carbon layer are formed at different distance from the arc discharge.

## Graphical abstract

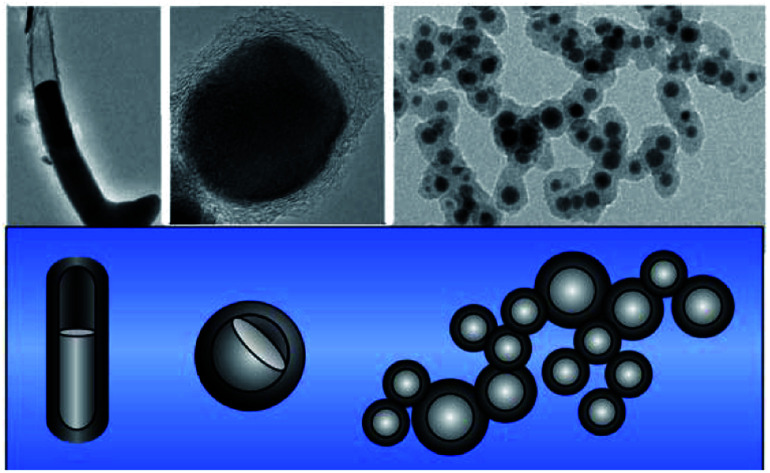

## 1. Introduction

An electric-arc method for the synthesis of nanostructured materials has been in widespread use since Kratschmer's pioneering work on the synthesis of fullerenes.^1^ He used a DC arc discharge between graphite electrodes in a helium medium. The work of Kratchmer was followed by cycles of work on the theoretical and experimental study of the kinetics of the formation of fullerenes in the arc discharge process.^2–4^ Under the conditions of synthesis, the stability of the discharge is determined by the thermal emission of electrons from the cathode, and only the anode is sprayed. Part of the sputtered material is deposited on the cathode and most of the sprayed material forms a stream of carbon vapour into the inert buffer gas.

Cooling in a buffer gas leads to the formation of a carbon structure that is deposited on a cooled screen. Depending on the synthesis conditions, carbon soot is formed on the screen, which can contain up to 15% fullerenes.^3^ The general concept of the mechanism of the formation of fullerene soot is the formation of a turbulent fan-shaped jet flowing from the interelectrode gap^4^ in which carbon condensation occurs through the forms of linear chains, cycles, and hemispheres.^2^ It is worth noting that the condensation of carbon vapour in the reaction gas medium changes the kinetics of condensation, which can lead to the formation of such structures as graphene.^5,6^

The use of a complex composition of electrodes, with the addition of other elements to graphite (mainly metals), gave a new direction to the development of electric arc synthesis as a result of which core–shell nanoparticles with a carbon shell and a core consisting of an additive element or its carbide phase can be formed. This direction was actively studied by the Saito^7^ and Majetich^8^ groups. In this case, the first group investigated the formation of a deposit on the cathode of the arc, and in the second there was formation of a composite material far from the arc on a cooled surface.

In the results of analyses of materials collected from the surface of a graphite cathode, it was concluded that the saturated vapour pressure of rare-earth elements determines the encapsulation of nanoparticles of these metals.^7^ Thus, the elements Y, La, Ce, Pr, Nd, Gd, Tb, Dy, Ho, Er, and Lu, having a low value of saturated vapour pressure, are encapsulated, and the elements Sm, Eu, Tm, and Yb, having a high value of saturated vapour pressure, are not encapsulated, with the exception of Tm. The model proposed by Saito consists of sputtering the composite electrode, which leads to the mixing of carbon vapours and metal vapours. The material begins to settle on the cathode. During the bombardment by high-energy particles from the interelectrode space, a carbon shell is formed. For most metals, as the particle temperature decreases, the solubility of carbon in this metal decreases, which can lead to the release of carbon onto the surface of the particle with the additional formation of a carbon shell, while the core crystallizes as a metal or its carbide phase.^7^ Note that metals with a high value of saturated vapour pressure do not condense at the cathode but instead fly away with the carbon vapour into the surrounding space.

For the formation of composite structures in a metal–carbon vapour jet, Madzhetich and Scott proposed a mechanism for the formation of metal–carbon structures deposited on the cold walls of the reactor.^8^ Cooling vapour in a buffer gas can lead to heterogeneous condensation and the formation of clusters of a liquid metal–carbon alloy. Starting from the nucleation point, the growth of the particles is determined by the concentrations of the components, the local temperature, the interaction potentials and the collision cross sections of all elementary components in the flow. The pressure of saturated metal vapours is one of the important parameters, which affects the formation of particles and determines the correlation with the results of Saito for rare-earth elements.

Other researchers^9^ who conducted experiments on arc sputtering of a carbon-composite material using 20 metals divided the materials into 4 categories: elements that are encapsulated as carbides (B, V, Cr, Mn, Y, Zr, Nb, Mo), elements that are not encapsulated (Cu, Zn, Pd, Ag, Pt), elements that form stable carbides at the molecular level and mix with carbon material (Al, Si, Ti, W), and iron group elements that initiate the growth of carbon nanotubes (Fe, Co, Ni). The authors noted that the Saito criterion associated with saturated vapour pressure may work for rare-earth elements, but not for alkaline ones, so B, V, Cr, Mn, and Y, which have a high value of saturated vapour pressure, are encapsulated. Based on their results, the authors postulate that the enthalpy of formation of specific metal carbides is important for the nanoparticle encapsulation process. If carbide does not exist, then encapsulation does not occur, although graphite nanocages may be observed in which the metal nanoparticle is located. Note that a strict separation of metals by the effect of their carbidization on the encapsulation process was not found. Therefore, Al, Si, and W form stable carbides but do not encapsulate, and Zr, which forms a stable carbide, is encapsulated.

Another group of researchers^10^ collected and ground the cathode deposit material using 15 metals (Ti, Cr, Fe, Co, Ni, Cu, Zn, Mo, Pd, Sn, Ta, W, Gd, Dy, Yb). The study of materials showed that the structure of some materials consists of metallic nanowires located in multi-walled carbon nanotubes, while in other materials such structures were not observed. The authors concluded that the number of electronic vacancies on the unfilled electron shells of metals is a key factor determining the formation mechanism of metal nanowires in carbon nanotubes. The basis for this conclusion was that Cr and Gd have the largest number of electronic vacancies, which leads to the growth of many of the longest metallic nanowires in carbon nanotubes.

Along with the volatility of metal vapours and the presence of its carbides, the formation of a nanostructured material is influenced by the molar content of the metal in the sprayed electrode, the pressure and the type of buffer gas, as well as the electrical characteristics of the arc discharge. So a method was proposed for controlling the size of iron nanoparticles by varying the composition of the buffer gas.^11^ The use of a mixture of He with a heavier Ar gas led to a decrease in the size of the nanoparticles. The use of pure argon led to an improvement in the crystallinity of the carbon material. In ref. 12, the authors analysed the formation of a carbon shell around copper nanoparticles and obtained hollow carbon spheres. According to their theory a carbon atoms are deposited on the Cu nanoparticles and form solid carbon shell. Different shrinking coefficients of C and Cu leads to crack of carbon shell and to escaping of the Cu nanoparticles. They expanded their theory to a number of other metals (Cu, Ag, Au, Zn, Fe, Ni). In the results, it was found that the addition of methane to helium leads to the “healing” of defects in the carbon envelope and the growth of the carbon envelope, as well as an increase in the size of the hollow carbon spheres. It has been proposed that the process of forming carbon shells covering metal nanoparticles depends on the boiling point and thermal expansion coefficient of metals, and copper provides the catalytic growth of a graphene structure on the surface of particles.

It should be noted that in the case of using carbon-containing gases, such as various hydrocarbons (methane, ethane, propane, acetylene *etc.*),^13–18^ vapours of alcohols and, carbon monoxide^19–22^ the working gas in the process of the arc-discharge decompose on the surface of the catalytic particle and is the source of carbon for the formation of solid structures. But in the early works^7–9^ the source of carbon was graphite, which evaporates under the influence of an arc discharge.

When co-sputtering carbon with metal, the metal also affects the formation of carbon structures. The use of elements of the iron group (Fe, Co, Ni) leads to the growth of carbon nanotubes.^23,24^ The growth mechanism of a nanotube in an arc discharge, proposed by the Saito group, is based on the phenomenon of co-evaporation of carbon and metal and cooling of a carbon–metal particle condensed from the gas phase where carbon saturation occurs and the carbon is released on the surface of a metal particle, thereby initiating nanotube growth.^25^ In a more developed form, this mechanism is called vapour–liquid–solid (VLS) and shows in more detail how the metal catalyst in the solid state poorly adsorbs molecules but how in the liquid state, it easily adsorbs molecules, which leads to a rapid catalyst oversaturation and the nucleation of carbon crystallization grains, while the growth of a nanotube begins with a liquid–solid interface.^26^

In addition to nanotubes and graphene, the formation of fishbone and bamboo-like structures is possible.^27–30^ The formation of carbon nanofibres is observed during thermal reforming of methane on Ni and Ni–Cu catalysts, where copper greatly influences the conversion of methane and the carbon yield. At various temperatures, carbon nanofibres of various shapes are formed, such as fishbone, lamellar, bamboo, branched, and on ionic carbon structures. In the process of methane reforming on the catalytic surface, depending on the characteristics of the active zones of the catalyst, either 6-membered or 5-membered rings are formed. After accumulating, the pentagons cause bending and deformation of the graphite plane, which leads to the formation of a bamboo structure. The model of the growth of the bamboo structure on the Ni particle consists of the carbon obtained in the reforming process being deposited on the nanoparticle and then it diffuses through the particle and forms an inner shell, which compresses the particle, forcing it to leave the shell. Periodic repetition of this process forms a bamboo structure.^29^ The bamboo structure with the same cavities and partitions indicates that the catalytic particle undergoes periodic processes of changes in shape.

The processes occurring during electric arc evaporation of various materials are diverse and lead to the formation of a large number of nanostructured forms with different properties.

At present, works related to the study of the mechanism of the synthesis of Sn–C nanomaterial in an electric arc is practically absent. However, there are a number of areas for the practical use of this material. First, it is used in the synthesis of new anode materials for Li-ion batteries with a large capacity for the capture of lithium ions.^31–36^ Second, the composite Sn–C material can be used as an effective temperature stabilizer in the region of tin melting. This direction is associated with the expanding scope of change phase materials in heat exchange processes.^37,38^

This paper conducted an experimental study of the Sn–C material synthesized by the plasma-arc method. The work examines the thermal, morphological and structural properties of the composite material. The obtained data allowed us to propose a physical model for the formation of a nanostructured Sn–C material.

## 2. Experimental

**Fig. 1:**
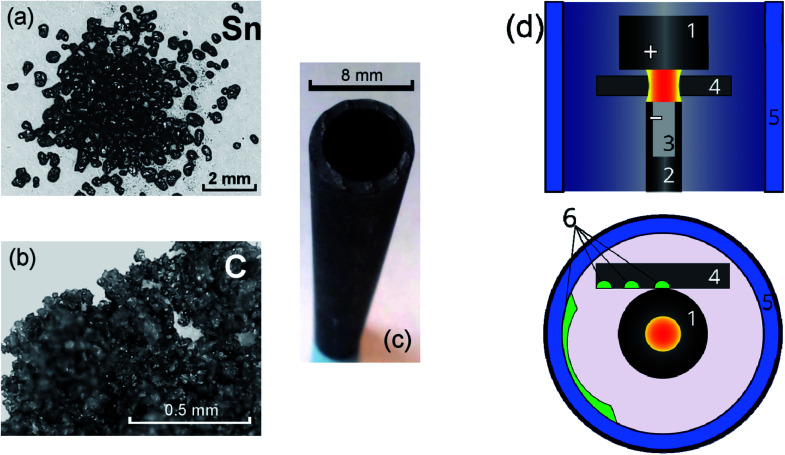
Initial powders of tin (a) and graphite (b) used for filling of the graphite anode (c). Principal scheme of the arc-discharge reactor (d).

**Fig. 2:**
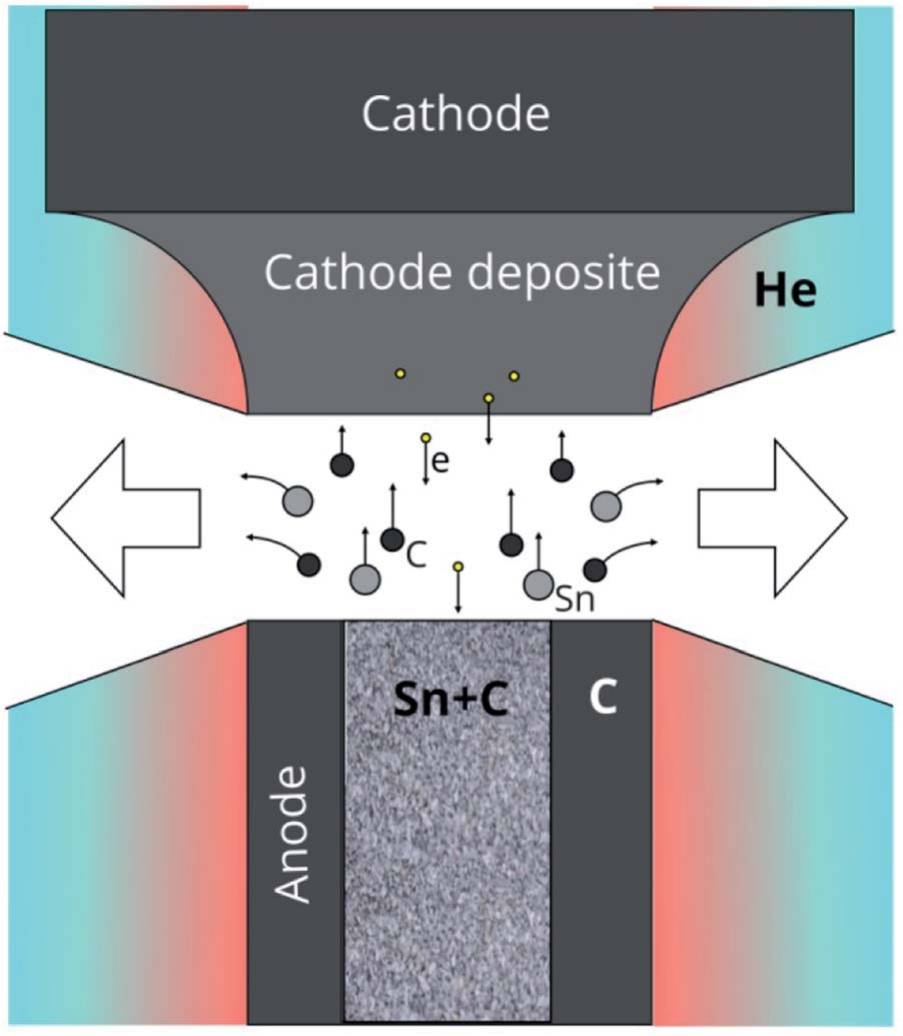
Process of the anode sputtering and fan-shaped jet formation.

A plasma-chemical synthesis of Sn–C nanomaterial was carried out using an electric arc reactor (Fig. 1(d)) consisting of a sealed vacuum chamber containing a cylindrical graphite cathode (1) with a diameter of 20 mm and a composite anode (2). The anode is a cylindrical graphite rod 8 mm in diameter with an axial cylindrical cavity 6 mm in diameter and 40 mm in depth (Fig. 1(c)). The cavity of the anode was tightly filled with a mixture of powders of tin (Fig. 1(a)) and graphite (Fig. 1(b)) with a weight ratio of 1/1 (3). The tin powder was produced by ACROS Organics Company in USA and has average granular size of 400 μm. The graphite for the anode, the cathode and the filling powder was produces by CATBOTEC Company in Russia. The average granular size of the graphite powder is 70 μm. The reactor chamber is pre-purged and filled with helium to a pressure of 12–50 torr. The source of direct current (Starke, Digital ARC250 ET, PRC) was used for ignition of arc discharge. For this, a voltage of 70 V was applied between the electrodes. An arc discharge of direct current 120–145 A was ignited between the electrodes by means of touching the electrodes together, that was determined by drop of the voltage to 5 V, and then subsequently moving of the electrodes apart to a distance (2 mm) providing a discharge voltage of 25 V. The arc discharge leaded to a thermal electron emission from the cathode. Electric field in the interelectrode gap accelerated electrons and high-energy electrons bombarded the anode. The anode was additionally heated by Joule heating and plasma irradiation. Evaporation, sputtering and consumption of the anode occurred, and maintaining a constant interelectrode distance and discharge voltage was carried out by moving the cathode. The products of the anode sputtering were partially deposited on the surface of the cathode, forming a cathode deposit, and the remainder get out from the interelectrode gap to the reactor chamber. In the reactor chamber the products of anode sputtering were mixed with the helium and formed a turbulent fan-shaped jet from the interelectrode gap (Fig. 2). Cooling leaded to the condensation of the products of the anode sputtering and nanomaterial production which was deposited on a water-cooled screen (5) maintained on 50 mm from the arc axis. The synthesized materials are collected from the screen and investigated by structural analysis methods.

To study the kinetics of formation of Sn–C structures, a graphite cylindrical rod with 50 mm length and 8 mm diameter was used as substrate (4) and was placed near the working electrodes, on the surface of which the material was deposited at different distances from the arc discharge. The materials were collected from the graphite substrate from areas located at 10–15 mm, 16–20 mm, 23–27 mm from the arc axis, which are marked green on the Fig. 1(d). To analyse the temperature distribution along the substrate, we used copper (*T*_w_ = 1085 °C) and chromel (*T*_w_ ∼1400 °C) wires with a cross-sectional area of 0.2 mm^2^ placed along the graphite substrate.

Structural studies were performed using transmission electron microscopy on a JEM-2010 (Japan, JEOLLtd) microscope, scanning electron microscopy in the modes of detecting secondary electrons on an SN-3400N (Hitachi) microscope, and X-ray diffraction analysis using a BrukerD8 Advance diffractometer with CuKα radiation (*λ* = 1.54 A). Thermophysical properties were studied using thermogravimetric calorimetric analyses using the device NETZSCHSTA 409.

## 3. Results and discussion

**Fig. 3:**
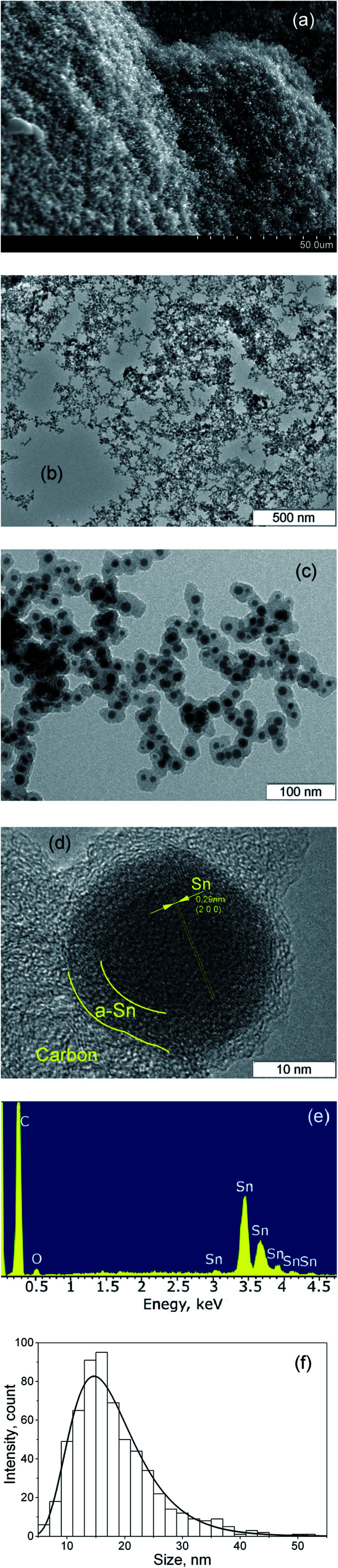
SEM image (a), and TEM images (b) and (c) of the synthesized material deposited on the cooled screen, HRTEM image of the synthesized core–shell Sn nanoparticle in the carbon matrix (d), EDX spectra of the synthesized material (e), size distribution of the Sn nanoparticles in the material (f).

**Fig. 4:**
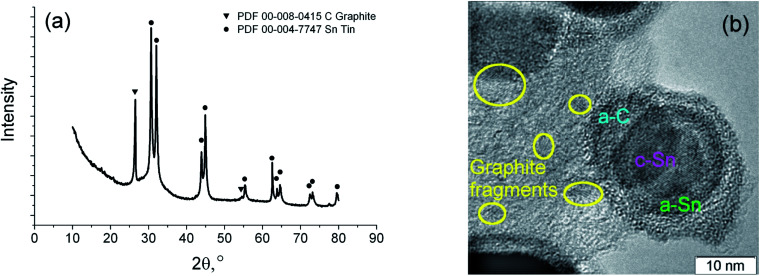
XRD spectrum of the synthesized material (a), HRTEM image of the core–shell nanoparticle with crystalline tin core, amorphous tin shell and amorphous carbon outer shell (b).

As a result of the arc discharge, the anode material is dispersed in the form of atomic and molecular components (Fig. 2). Electrode sputtering products (Sn + C) are partially deposited on the cathode, forming a cathode deposit, and the remaining mass leaves the interelectrode space, forming a turbulent fan-shaped jet.^2^ The temperature in the jet decreases with the distance from the discharge gap and from the central section to the jet boundaries. The cooling of the carbon and tin vapours leads to condensation and agglomeration processes. The resulting material is deposited on a cooled screen in the form of a loose carbon–tin layer (Fig. 3(a)). As EDX analysis showed, the material consists only of tin and carbon (Fig. 3(e)). The collection and treatment by ultrasound of the material in the preparation process for TEM studies leads to the destruction of the macrostructure of the layer. The material takes the form of branched chains of particles (Fig. 3(b and c)). The individual particles are tin spherical particles surrounded by a layer of carbon with weak signs of graphitization. However, XRD analysis (Fig. 4(a)) shows the presence in the material of the graphite structure and the structure of a tin *I*4_1_/*amd* symmetry group. The graphite structure is presented in forms of the tiny graphite fragments in the carbon matrix (Fig. 4(b)). The size distribution of the tin particles is described by a log-normal distribution with an average size of 18 nm (Fig. 3(f)). Some Sn particles have a core–shell structure (Fig. 3(d)). It is assumed that the core–shell structure consists of a crystalline core and an amorphous shell, the average thickness of which is 3 nm. Other Sn particles are composed entirely of crystalline tin, and completely amorphous tin particles are also possible.

Annealing the material in an oxygen-containing atmosphere for 2 hours at 800 °C leads to complete oxidation of both tin and carbon. The oxidation products are solid SnO_2_ and CO_2_ gas. The mass of the solid residue allowed us to establish that the original material contains 34 wt% Sn.

**Fig. 5:**
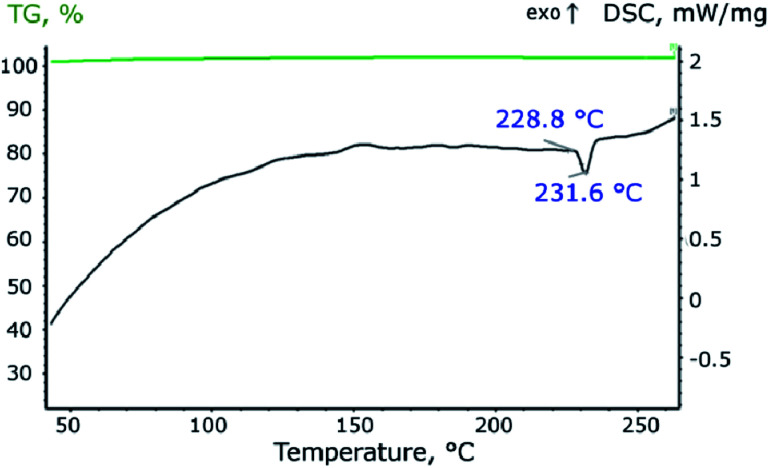
Thermogravimetric and calorimetric analysis of the synthesized material.

In the process of heating the material in an inert atmosphere at a temperature of 228.8 °C, heat absorption begins, which corresponds to the process of melting tin (Fig. 5). As seen, the melting point of the nanosized tin is slightly lower than the melting point of the bulk tin.^39,40^ Integration of the peak makes it possible to estimate the values of the enthalpy of melting, which is 4.7 J g^−1^. Taking into account that the mass content of tin in the material is 34%, and the reference value of the melting enthalpy of bulk tin is 59.55 J g^−1^, the theoretical melting enthalpy of the synthesized material should be 20.25 J g^−1^, which is almost four times more than experimentally measured. A lot of papers predict that the enthalpy of melting of Sn nanoparticles depends on both shape and size. For example in ref. 41 authors calculated the entropy of 20 nm Sn spherical particles which is about 3% less than for bulk tin. In ref. 42 enthalpy of melting tin nanoparticles was about 10% less for 20 nm size and about 40% less for 5 nm size than for bulk material. And other authors declared about 30% decrease for 20 nm Sn nanocrystals related to bulk.^43,44^ However the enthalpy didn't exceed 30% diminution for 20 nm tin nanoparticles and 60% for 5 nm tin nanoparticles. In the present study the enthalpy exceed about 75% diminution for 18 nm Sn nanoparticles. These effects may be because part of the tin in the material is in an amorphous state, the melting process of which begins at a lower temperature and occurs with a lower melting enthalpy.^45^

**Fig. 6:**
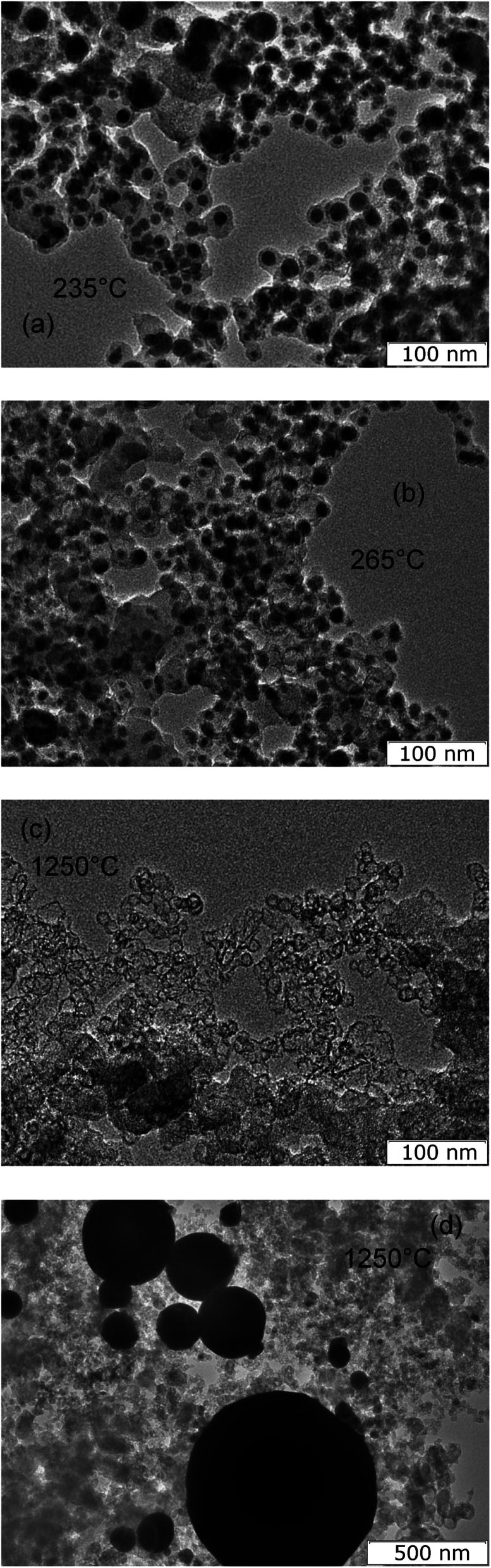
TEM image of material heat-treated at 235 °C (a), at 265 °C (b), at 1250 °C (c and d).

Herewith the material retains its structure when heated up to the melting point and even slightly higher (235 °C) (Fig. 6(a)). During melting, the density of tin changes dramatically from 7176.1 kg m^−3^ to 6982.2 kg m^−3^,^46^ which corresponds to a sharp change in volume of 2.8%. In this case, it is assumed that part of the tin particles break the carbon shell, the thermal expansion of which is significantly less,^47,48^ and escape from it, leaving empty shells behind. Such structures begin to appear in the material heated to a temperature of 265 °C (Fig. 6(b)) and prevail in the material heated to 1250 °C (Fig. 6(c)). At the same time, liquid tin is merged into droplets with sizes of 100–500 nm (Fig. 6(d)).

However, the processes in arc discharge reactor leading to the formation of the spherical tin nanoparticles encapsulated in the carbon matrix remain unclear. Saito^7^ proposed that arc discharge leads to formation of hot metal nanoparticles which are saturated by carbon. Then the nanoparticles are cooled and saturated carbon release on the particle surface in form of solid carbon shell. Nevertheless tin has very low solubility of carbon.^49^ And cooling of a 20 nm nanoparticle potentially gives a carbon shell less than 1 atom thickness. So the Saito model couldn't explain formation of the thick carbon shell around the tin nanoparticles found in the synthesized material. Majetich and Scott^8^ postulated that encapsulated metal nanoparticles were formed by heterogeneous condensation of metal and carbon vapors with formation of metal–carbon alloy. However carbon has low wettability by tin and it couldn't create uniform alloy.^50^ So the Majetich model couldn't explain the formation of the Sn nanoparticles in the carbon shell. Seraphine^9^ explained a formation of metal nanoparticles encapsulated in carbon by enthalpy of metal carbide formation, so if metal has carbide form, it is capsulated in carbon. But tin has not any carbide form. So the Seraphine criteria couldn't be applied to the formation of the Sn nanoparticles encapsulated in carbon presented in the present study.

To study the formation of the structure, materials were collected from various distances from the axis of the arc discharge. Materials deposited on a graphite substrate at different distances have characteristically different structural features.

**Fig. 7:**
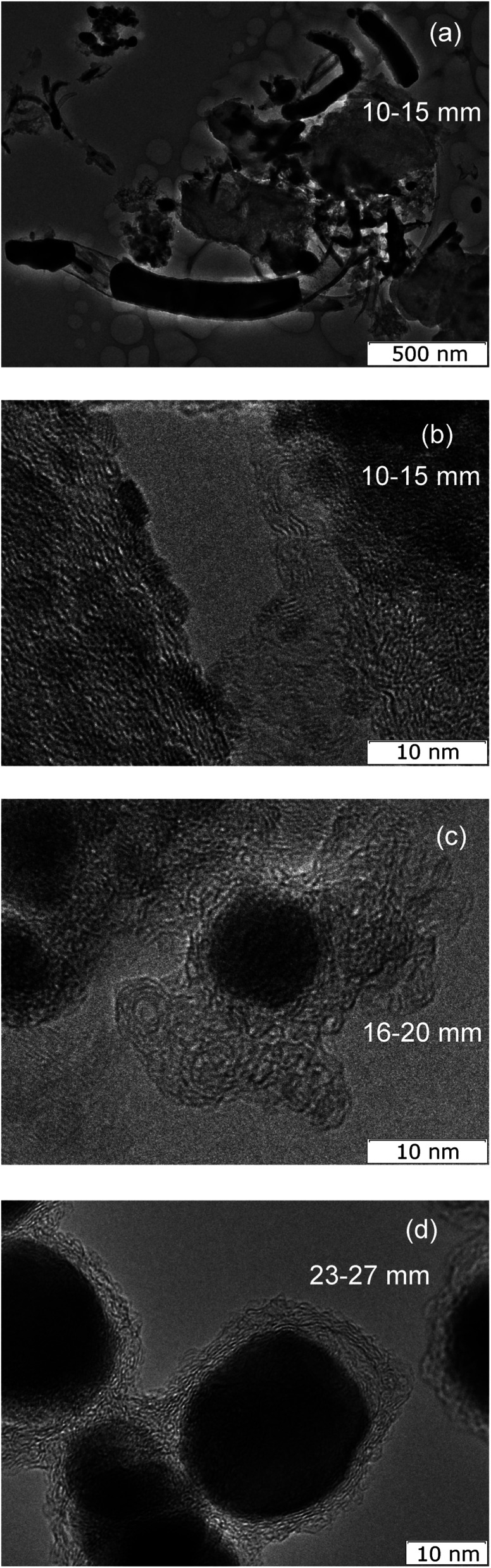
TEM (a) and HRTEM images of material collected from areas 10–15 mm from the arc discharge, 16–20 mm (c) and 23–27 mm (d).

The material collected from the region at a distance of 10–15 mm contains elongated carbon shells, partially filled with tin (Fig. 7(a)). The transverse diameters of such particles range from 30 to 200 nm with lengths from 50 nm to 2 μm. The thickness of the carbon envelope usually thins from one end to the other. On some particles, on the outer side of the carbon shell there are tin crystals of 2–3 nm (Fig. 7(b)).

The material collected from the graphite substrate from a region located at a distance of 16–20 mm from the discharge axis contains both elongated Sn–C structures and spherical Sn nanoparticles with dimensions of approximately 10 nm, covered with a carbon shell. Moreover, this carbon shell has a branched structure (Fig. 7(c)).

In the material collected from the area of the graphite substrate at a distance of 23–27 mm from the discharge axis, elongated Sn–C structures are no longer observed. The material consists of carbon particles ∼20 nm with a spherical cavity filled with 90% crystalline tin and 10% void (Fig. 7(d)).

**Fig. 8:**
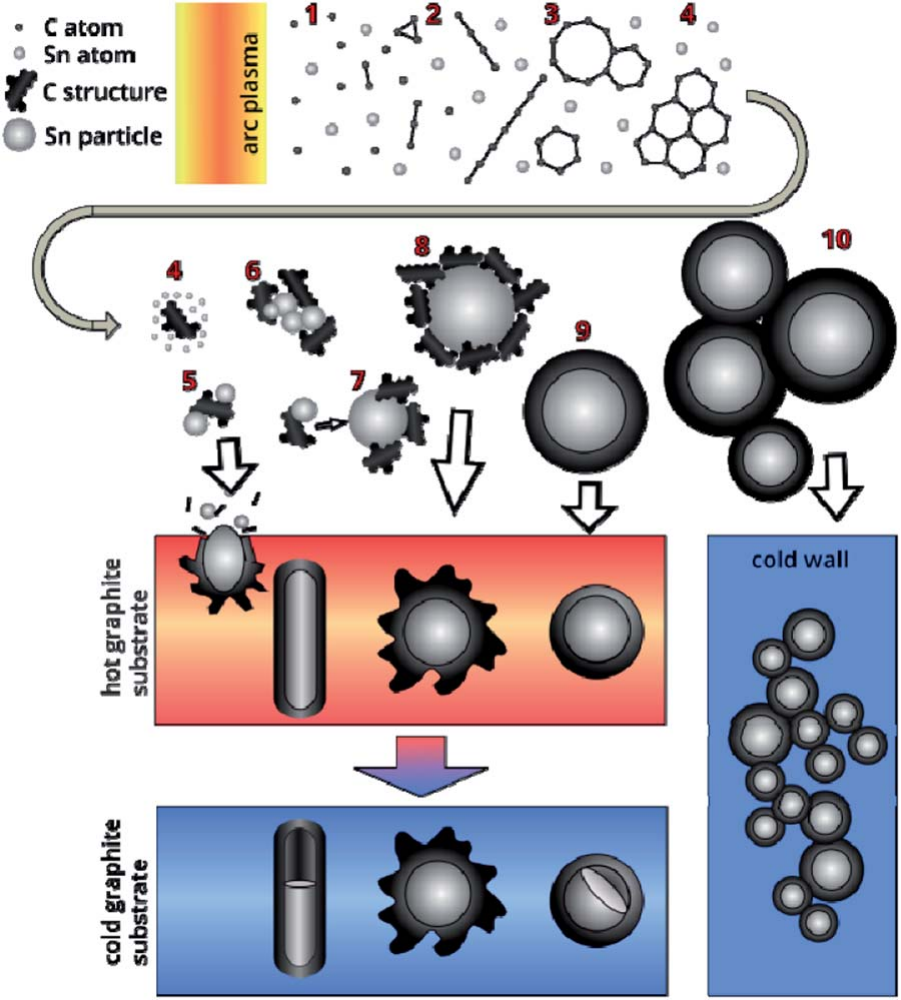
Scheme of Sn–C material formation during arc discharge.

The presence of such structures gave reason to suggest new mechanism for the formation of the Sn–C nanomaterial (Fig. 8). The evaporation rate of the graphite part of the anode, using the Langmuir evaporation model,^51^ allows for estimation of the temperature at the anode surface, which is ∼3900 K. The composition of the gas-plasma mixture at this temperature was calculated using CEA NASA software using the principle of minimum Gibbs energy, the condition of quasi-neutrality and the law of mass conservation, where the initial concentrations of the components correspond to the experimental conditions.^52^ According to the calculations, the composition of the gas includes atomic components: He 78.6 mol%, C 14.1%, Sn 2.3%, dimer C_2_ 2.5%, and trimer C_3_ 2.4%; the remaining components are less than 0.1% including electron gas at 0.03%. When a gas mixture flows out of the discharge gap, a fan-shaped jet is formed, the temperature of which decreases as it moves from the discharge and from the central section of the jet to the jet boundaries. As the vapours enter from the hot areas (stage 1 in Fig. 8) into the colder areas, the following processes occur: first, carbon begins to condense, passing through the stages of the formation of fullerene soot. The kinetics of carbon condensation involve the formation of polyatomic chains (stage 2) and cycles (stage 3), which are nuclei of the fullerene structure. However, the formation of a closed spherical carbon structure of fullerene preferably occurs in a helium atmosphere, while the presence of other gases adversely affects the yield of fullerenes.^6^ Accordingly, the presence of tin vapour in the carbon condensation zones leads to the formation of structures including disordered six- and five-membered carbon rings, which are nuclei of an amorphous and partially graphitized carbon structure (stage 4). Unlike soot formation in flames,^53,54^ where PAH (poly-aromatic hydrocarbons) are formed, in an arc discharge in an inert atmosphere in hot areas, all carbon structures have many dangling bonds that are mobile and have many degrees of freedom. Permanent rebuilding of the carbon structure occurs, and existing bonds can break and form new, flat structures can bend, flip, and fold.^55^ Then, at temperatures below 2200 K, the condensation of tin vapour begins. Existing fragments of carbon structures can act as tin condensation centres, forming Sn–C clusters (stage 5). Agglomeration of such clusters (stage 6) leads to the merging of tin drops, while condensed carbon remains on the surface of the drop (stages 7, 8). In this case, the disordered carbon structure retains a large number of dangling bonds, with the result that further cooling of the particles leads to a reorganization of the carbon structure into the form of a solid shell around the tin particle (stage 9). Collisions and agglomeration of particles formed in stages 8–9 leads to the formation of chains of particles (stage 10). The soft carbon shell is reduced along with the thermal shrinking of the tin liquid drop and a decrease accompanying the liquid–solid phase transition.^46^ The removal of heat through contact with the walls of the reactor leads to the fact that the carbon structure stabilizes and forms a rigid shell. The cooling of the liquid tin particle encapsulated by carbon occurs from the tin–carbon contact surface, leading to the formation of an amorphous tin layer. The solidification of the amorphous tin layer occurs with the release of heat, which leads to a slowdown in the cooling of the core and its crystallization.

**Fig. 9:**
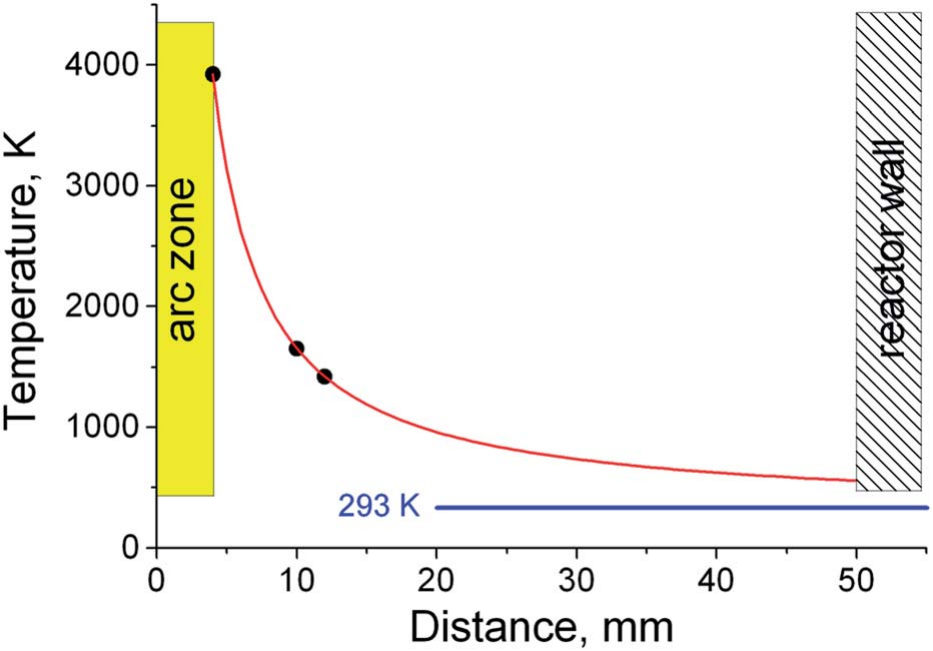
Temperature distribution in the jet.

In the case of deposition of electrode sputtering products on a nearby graphite substrate with a high heat capacity, rapid quenching of the Sn–C structure occurs. As was established earlier, the temperature at the surface of the anode is ∼3900 K. At an infinite distance, the temperature should not differ from the temperature of the surrounding space (293 K). Taking into account these conditions, as well as approximating the experimental data on the temperature distribution in the formed gas-dynamic jet^56^ using reference metal samples with different melting points, we estimated the temperature values in various areas (Fig. 9).

At a distance of 10 mm, corresponding to the smallest distance from the discharge to the surface of the graphite substrate, the temperature does not exceed 1650 K. At such temperatures, tin condensation and the formation of Sn–C clusters and their agglomerates are possible (stages 5–6). Getting to the surface of the graphite substrate, the Sn–C clusters are cooled, merge and form a tin drop covered with a disordered carbon structure. Further bombardment of such a structure by the incoming Sn–C clusters can lead to a local destruction of the carbon structure on the surface and the merging of liquid tin. The mass of tin, which increases during the discharge process, initiates its outflow through the destroyed shell, while the carbon coming in with the tin forms a shell around the leaky tin drop. This process contributes to the formation of an elongated shape of a tin core coated with a carbon shell. The formation of a similar structure on the surface of a graphite substrate, which has a high heat capacity, leads to stabilization and an increase in the rigidity of the carbon shell. Gradual cooling after the discharge is turned off leads to compression of the tin due to thermal reduction of the volume and a phase transition, which leads to the formation of unfilled voids inside the elongated carbon shell.

At a distance of approximately 16 mm, corresponding to a temperature of ∼1100 K, a tin liquid particle is formed with a shell of a disordered carbon structure. The rapid cooling of such a structure leads to the formation of a tin particle with a branched carbon shell. When cooled to room temperature, the shell shrinks together with the tin particle.

At distances of approximately 23 mm (*T* ∼850 K), the processes of forming a continuous carbon shell have already taken place and liquid drops of tin coated with soft carbon are deposited on a graphite rod. Cooling the structure to the graphite substrate temperature leads to a partial graphitization of the carbon shell, there by giving it rigidity. The gradual cooling to room temperature of the rod and the structures deposited on it leads to a decrease in the volume of liquid tin and the formation of a void inside the carbon envelope due to different coefficients of thermal expansion of tin and carbon, as well as a volume reduction of tin during the phase transition (505 K).^46^ Statistical estimates have shown that the volume of the voids is 5.2% of the cavity inside the carbon shell. Given the thermal contraction of carbon, such a decrease in volume is characterized by a change in the density of the tin from 6.85 to 7.29 g cm^−3^, corresponding to temperatures of 675 K and 293 K (∼20 °C).

## 4. Conclusion

As a result of electric arc sputtering of the composite Sn/C anode, a gas non-isothermal heterogeneous fan-shaped jet is formed, which flows from the interelectrode gap into the space of the reactor chamber. Heterogeneous condensation of carbon and tin vapour in the jet leads to the processes of condensation of the carbon structure, condensation of tin drops on carbon particles, agglomeration of tin–carbon particles and coagulation of tin with the formation of spherical tin drops, covered with a carbon sheath, and agglomeration of similar structures and crystallization of liquid tin nanoparticles. As a result, a material, which is a spherical tin nanoparticle packed in a carbon matrix, is deposited on a water-cooled screen. The average size of the tin nanoparticles is 18 nm. Some nanoparticles have a core–shell structure with a crystalline core and an amorphous tin shell formed due to different rates of tin solidification. This structure reduces the enthalpy of the material melting by four times.

## Conflicts of interest

There are no conflicts to declare.

## Supplementary Material
